# Cigarette smoke extract exacerbates hyperpermeability of cerebral endothelial cells after oxygen glucose deprivation and reoxygenation

**DOI:** 10.1038/s41598-019-51728-2

**Published:** 2019-10-30

**Authors:** Ashton Bernard, Jacqueline M. Ku, Ross Vlahos, Alyson A. Miller

**Affiliations:** 1 School of Health and Biomedical Sciences, RMIT University, PO Box 71, Bundoora, VIC 3083 Australia; 2 Institute of Cardiovascular & Medical Sciences, British Heart Foundation Glasgow Cardiovascular Research Centre, University of Glasgow, Glasgow, G12 8TA United Kingdom

**Keywords:** Cardiovascular diseases, Cardiovascular diseases, Acute inflammation, Acute inflammation

## Abstract

Cigarette smoking is a risk factor for stroke and is linked to stroke severity. Previous studies have shown that cigarette smoke extract (CSE) triggers endothelial dysfunction *in vitro* by initiating oxidative stress and/or an inflammatory response. In addition, cerebral endothelial dysfunction (particularly at the level of the blood-brain barrier [BBB]) contributes to stroke pathogenesis. Therefore, we hypothesized that cigarette smoking may influence stroke, at least in part, by exacerbating ischaemia-induced BBB disruption. To test this, we examined the effect of CSE on the permeability of cerebral endothelial cells exposed to oxygen glucose deprivation and reoxygenation (OGD + RO). We found that the loss of BBB integrity following ischaemic/reperfusion-like conditions was significantly worsened by CSE. Despite this being associated with increased mRNA expression of Nox catalytic subunits, reactive oxygen species (ROS) levels were however markedly lower. Furthermore, this occurred in association with elevated expression of antioxidant enzymes (SOD1, SOD2, and Gpx-1), suggesting an antioxidant defence response. Lastly, we found that CSE significantly upregulated mRNA expression of cytokines (IL-6 and TGF-β). Collectively, these results show that acute exposure to CSE worsens BBB disruption caused by OGD + RO, however, this is not linked to elevated ROS levels but may involve inflammatory mechanisms.

## Introduction

Cigarette smoking is a major cause of morbidity and mortality worldwide^1^. In addition to causing various cancers^2^, cigarette smoking is linked to the pathogenesis of a number of diseases that affect the brain. For example, smoking is a risk factor for ischaemic and haemorrhagic stroke^3^, and silent cerebral infarction^4^. Cigarette smoking is also a major cause of chronic obstructive pulmonary disease (COPD), accounting for more than 95% of cases in industrialized countries^5^. Recent studies have shown that strokes are more prevalent in COPD patients compared with the general population^6–8^, and clinical studies have revealed that COPD is linked to worse stroke outcomes including mortality^9,10^. Given smoking is an established risk factor for stroke and is linked to worse stroke outcomes the association between COPD and stroke may be largely dependent on exposure to cigarette smoke^6^.

Cigarette smoke contains over 4,000 chemicals including nicotine and reactive oxygen species (ROS, e.g. superoxide, hydrogen peroxide)^11^. These oxidants give rise to secondary ROS by inflammatory cells within the lung as part of an inflammatory-immune response towards a pathogen or irritant^6^. These inflammatory cells also have an impaired phagocytic function, resulting in impairment in clearance of apoptotic cells, contributing to the chronic inflammatory state in the lungs and leading to an ongoing cycle of damage and remodelling in the airways and lung tissue^12,13^. In addition to triggering local oxidative stress and inflammation in the lungs, the chemicals found in cigarette smoke can pass through the lung and initiate oxidative stress and inflammation in cells remote from the primary site of exposure^14,15^. At the level of the cerebral vasculature this can in turn trigger cerebral endothelial dysfunction^6,16^. Indeed, exposure of rats to cigarette smoke impairs endothelial-dependent vasodilator responses of cerebral arterioles *in vivo* by activating the Nox-NADPH oxidases^17^, ROS generating enzymes that are major contributors to cerebral endothelial dysfunction in numerous disease states including stroke^16,18^. Furthermore, using cigarette smoke extract (CSE) to mimic physiological concentrations of heavy smokers, several studies have shown that cigarette smoking triggers blood-brain barrier (BBB) disruption *in vitro* via oxidative and inflammatory mechanisms^19,20^. Given cerebral endothelial dysfunction, particularly at the level of the BBB, is implicated in stroke pathogenesis^21^, it is conceivable that cigarette smoking may influence stroke, at least in part, by exacerbating ischaemia-induced BBB disruption. Therefore, the aim of this study was to examine whether CSE worsens BBB disruption using a well-established *in vitro* BBB stroke model, and to determine whether this is associated with elevated ROS production and/or inflammation.

## Methods

### Cigarette smoke extract (CSE) preparation

CSE was prepared as previously described^22^. Briefly, this involved using one filtered Winfield Original Red cigarette (1.2 mg of nicotine, 16 mg of tar, 15 mg of CO). The cigarette was lit and using a 30 ml syringe cigarette smoke was bubbled (flow rate of 3 mL/second) into 25 mL of culture media (Dulbecco’s modified Eagle’s medium [DMEM] media). This process was repeated until the cigarette had burned through just prior to the filter. The resultant solution was defined as 100% CSE. 100% CSE was then filtered before being diluted in media. CSE was utilised within 15–30 minutes after preparation.

### Culture of mouse cerebral microvascular endothelial cells

Mouse microvascular cerebral endothelial cells (bEnd.3 cells; ATCC CRL-2299) were grown in DMEM media (containing 10% fetal bovine serum [FBS]) at 37 °C in a humidified 5% CO_2_ atmosphere^23^. Cells were passaged every 3–4 days. Culture media was changed after 24 h of passaging and every 2 days thereafter. Experiments were performed with cells from passages 26 to 34.

### Oxygen glucose deprivation (OGD) and reoxygenation (RO) of bEnd.3 cells

**Figure 1:**
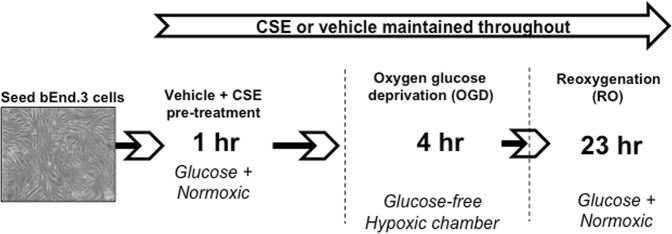
Flow chart depicting Oxygen glucose deprivation (OGD) and reoxygenation (RO) of bEnd.3 cells. Two days post-confluent bEnd.3 cells were pre-treated with either media alone (vehicle) or CSE (5, 10, 20 or 40%) for 1 hour. Cells were then incubated for 4 hours in a hypoxic chamber in either glucose-free media containing CSE or OGE alone (vehicle). followed by oxygen glucose deprivation (OGD) with glucose-free vehicle or CSE for 4 hours in a hypoxic chamber. After 4 h of OGD, media was replaced with glucose-containing media (oxygenated with air) for a further 23 h incubation at 37 °C (5% CO_2_ atmosphere) (Fig. 1). For each OGD + RO experiment, time-controlled normoxic controls were run alongside by incubating cells for 27 h in CSE diluted in glucose-containing media or media alone (vehicle).

bEnd.3 cells were seeded at a density of 7 × 10^4^ cells/cm^2^ in 96-well plates or T75 tissue culture flasks (Greiner Bio-One), or at 4 × 10^4^ cells/well in tissue culture inserts (translucent polyethylene terephthalate [PET], 0.4 μm pore size; Greiner Bio-One) and grown to confluence. Two-days post-confluent cells were washed twice with DMEM glucose-containing media to remove culture media, containing FBS, and replaced with either CSE (5%, 10%, 20% or 40%) diluted in DMEM glucose-containing media or media alone (vehicle). Cells were incubated for 1 h at 37 °C (5% CO_2_ atmosphere), washed twice with DMEM glucose-free media pre-equilibrated in OGD gas mixture for 5 mins (95% N_2_ and 5% CO_2_). Cells were then incubated for 4 h in a humidified hypoxia chamber (Biospherix, Lacona, USA; 95% N_2_, 5% CO_2_) in either OGD media containing CSE (5, 10, 20 or 40%) or OGD media alone (vehicle) (Fig. 1)^23^. A digital oxygen controller maintained the oxygen level at 0.3% and CO_2_ at 5% for the duration of the experiment. After 4 h of OGD, media was replaced with either vehicle or CSE (5%, 10%, 20% or 40%) diluted in glucose-containing, serum-free DMEM media (oxygenated with air) for a further 23 h incubation at 37 °C (5% CO_2_ atmosphere) (Fig. 1). For each OGD + RO experiment, time-controlled normoxic controls were run alongside by incubating cells for 27 h in CSE (5%, 10%, 20% or 40%) diluted in glucose-containing, serum-free DMEM media or media alone (vehicle) at 37 °C (5% CO_2_ atmosphere).

### Measurement of cell viability of bEnd.3 cells

Morphological changes in bEnd.3 cells were assessed using phase contrast imaging. Briefly, bEnd.3 cells were seeded into 12-well plates and exposed to either normoxic or OGD + RO conditions, as described above, and treated with either vehicle or CSE (5, 10, 20 or 40%). Cells were then imaged using a microscope (Nikon Eclipse TS100, Japan) at ×100 magnification and image capture camera (Infinity 3 Lumen*era*, Canada). To measure cell viability a 3-(4,5 dimethylthiazol-2-yl)-5-(3-carboxymethoxyphenyl)-2-(4-sulfophenyl)-2H-tetrazolium salt (MTS) assay (Promega, Australia) was performed as previously described^23^. bEnd.3 cells were seeded into clear 96-well plates (Greiner, Bio-One) and exposed to OGD + RO or normoxic conditions with either vehicle or CSE (5, 10, 20 or 40%) treatment, as described above. Following RO, bEnd.3 cells were washed twice with pre-warmed Krebs-HEPES buffer [composed of (in mmol/L) 99 NaCl, 4.7 KCl, 1.2 MgSO_4_, 1.0 KH_2_PO_4_, 19.6 NaHCO_3_, 11.2 Glucose 20 Na-HEPES and 2.5 CaCl_2_, pH = 7.4] prior to incubating with the MTS solution for 1 h at 37 °C (5% CO_2_). A BMG Clariostar plate reader (BMG Labtech, Germany) was used to measure absorbance at a wavelength of 490 nm.

### Measurement of paracellular permeability of bEnd.3 cells

Paracellular permeability was assessed by measuring the diffusion of 70 kDa fluorescein isothiocynate (FITC)-dextran (Sigma) across monolayer cultures of bEnd.3 cells, as previously described^23,24^. Tissue culture inserts (0.4 μm pore size; Greiner Bio-One) were fitted into 24-well culture plates (Greiner Bio-One) and then pre-soaked with culture media in both the upper ‘apical’ chamber and lower chamber 1 h prior to seeding. bEnd.3 cells were subsequently seeded into the upper chamber in culture media as above. Culture media was replaced at this time and after 24 hours. Two-days post-confluent, bEnd.3 cells were exposed to OGD + RO or normoxic conditions with either vehicle or CSE (10%) treatment, as described above. Following RO, pre-warmed Krebs-HEPES buffer was replaced in the lower chamber of fresh wells and inserts transferred into these wells. Both the upper and lower chambers were then washed with pre-warmed Krebs-HEPES buffer twice and then FITC-dextran (1 mg/mL) was added to the upper chamber and left to incubate for 1 h at 37 °C (5% CO_2_). Duplicate samples from the lower chamber were removed and transferred into a black 96-well plate (PerkinElmer, Australia). Using a BMG Clariostar plate reader, fluorescence intensity was measured at an excitation wavelength of 492 nm and emission wavelength of 518 nm.

### Measurement of superoxide levels in bEnd.3 cells

bEnd.3 cells were seeded into a white 96-well plate (PerkinElmer) and exposed to OGD + RO or normoxic conditions with either vehicle or CSE (5, 10, 20 or 40%) treatment, as described above. Following RO, L-012 (100 μmol/L)^24,25^ was added to each well and photon counts measured using a BMG Clariostar plate reader (45 cycles, 3 seconds per well).

### Measurement of hydrogen peroxide production by bEnd.3 cells

bEnd.3 cells were seeded into a black 96-well plate (PerkinElmer) and exposed to OGD + RO or normoxic conditions with either vehicle or CSE (10%) treatment, as described above. Cells were treated with 15 μmol/L Amplex Red^®^ reagent (10H-Phenoxazine-3,7-diol, 10-acetyl- 119171-73-2) and 0.1 U/mL horseradish peroxidase (HRP) (Thermofisher Scientific)^25^. Amplex red and HRP were also added to hydrogen peroxide standards (0.000, 0.078, 0.156, 0.312, 0.625 and 1.25 μmol/L). Fluorescence was then measured using a BMG Clariostar plate reader (121 cycles, 40 s per well) at emission filter 590 nm and excitation filter 530 nm.

### RT-PCR

bEnd.3 cells were seeded into T75 cell culture flasks (Greiner Bio-One). Two-days post-confluent bEnd.3 cells were exposed to OGD + RO or normoxic conditions with vehicle or CSE (10%) treatment, as described above. Following RO, flasks were washed with 0.01 M phosphate buffered saline (PBS) twice and cells scraped off with 0.01 M PBS. RNA was extracted using the Qiagen RNase-free DNase kit (Qiagen) as per manufacturer’s instructions. Both the purity and yield of RNA were quantified using a Nanodrop 2000 spectrophotometer (Thermofisher Scientific). cDNA was synthesised using a QuantiTect Reverse Transcription kit (Qiagen). QuantiFast SYBR® Green primers were used to measure Nox1 (NM_172203), Nox2 (NM_007807) and Nox4 (NM_015760), SOD1 (NM_011434), SOD2 (NM_013671), SOD3 (NM_011435), Gpx-1 (NM_008160), and the housekeeping gene 18S (NR_003278). TaqMan^®^ Universal Master Mix (Applied Biosystems) and mouse-specific TaqMan^®^ Gene Expression assays (Applied Biosystems) were used to measure IL-4 (NM_021283.3), IL-1β (NM_008361.3), TNF-α (NM_013693.3), IFN-γ (NM_008337.3), IL-6 (NM_031168.1), TGF-β (NM_011577.1) and the housekeeping gene 18S (NR_003278.3) via a QuantStudio^TM^ 7 Flex Real-Time PCR System (Applied Biosystems). Data were normalized to ribosomal 18s and represented relative to expression levels in normoxic vehicle control samples using the 2^−ΔΔCt^ method^26^.

### Data and statistical analyses

All statistical analysis was performed using GraphPad Prism 6 (GraphPad Software, Version 6.07, La Jolla, CA, USA). Results are represented as mean ± SEM and a *P* < 0.05 was considered to be statistically significant. Normoxic groups were compared using a Student’s unpaired t-test. A one-way ANOVA with a Bonferroni post-hoc test was performed for OGD + RO groups for FITC-dextran passage, cell viability, superoxide production, hydrogen peroxide production, and mRNA expression.

## Results

### Effect of CSE on cell viability of bEnd.3 cells

**Figure 2:**
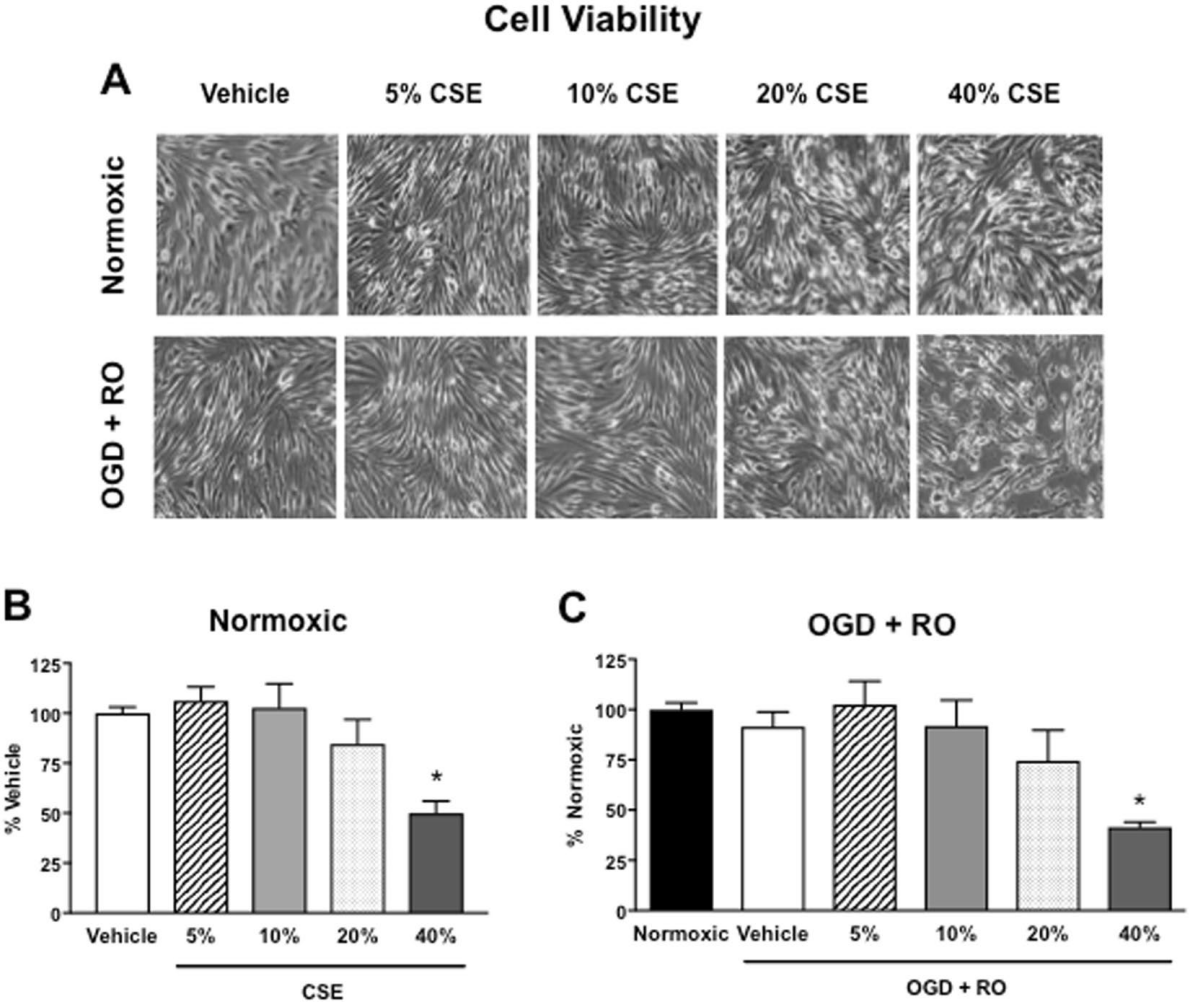
Effect of cigarette smoke extract (CSE: 5, 10, 20, and 40%) on the morphology and cell viability of bEnd.3 cells exposed to normoxic or OGD + RO conditions. Phase contrast images of normoxic (top panel) and OGD + RO (bottom panel) bEnd.3 cells (representative of n = 3, **A**). Quantification of cell viability of normoxic (**B**) and OGD + RO (**C**) bEnd.3 cells. Results are represented as of vehicle-treated cells and are given as mean ± SEM (n = 6–8). *P < 0.05 vs. vehicle-treated cells (One-way ANOVA with Bonferroni post-hoc test).

Treatment of normoxic or OGD + RO bEnd.3 cells with either 5 or 10% CSE resulted in no obvious morphological changes compared with vehicle-treated cells (Fig. 2A). However, 20% or 40% CSE resulted in a loss of their typical spindle-shape appearance as well as visible cell retraction (Fig. 2A). 5, 10, or 20% CSE had no significant effect on the viability of either normoxic or OGD + RO bEnd.3 cells relative to vehicle-treated cells (Fig. 2B,C, *P* > 0.05, n = 6–8), whereas 40% CSE decreased cell viability by ~50% (Fig. 2B,C, *P* < 0.05).

### Effect of CSE on paracellular permeability of bEnd.3 cells

**Figure 3:**
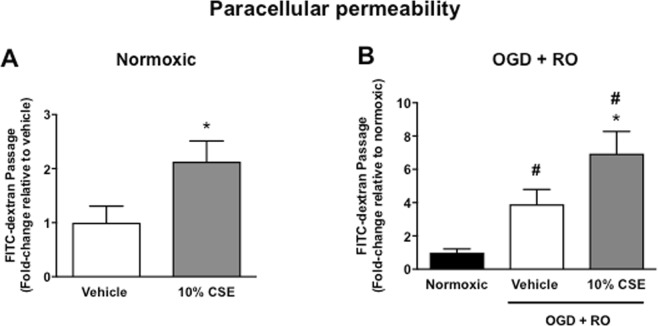
Effect of cigarette smoke extract (CSE: 10%) on the paracellular permeability of bEnd.3 cells exposed to normoxic (**A**) or OGD + RO conditions (**B**). Results are expressed as fold-change in FITC-dextran passage relative to either vehicle-treated cells (**A**) or normoxic controls (**B**) and are given as mean ± SEM (n = 6–8). *P < 0.05 vs. vehicle-treated cells (A, Student’s unpaired t-test; B, ^#^*P* < 0.05 vs. normoxic controls (One-way ANOVA with Bonferroni post-hoc test).

Treatment of normoxic bEnd.3 cells with 10% CSE significantly increased FITC-dextran passage (~2 fold) and thus endothelial cell paracellular permeability, relative to vehicle-treated normoxic cells (Fig. 3A, *P* < 0.05, n = 6–8).

Exposure of bEnd.3 cells to OGD + RO significantly increased paracellular permeability (~4-fold) relative to normoxic controls (Fig. 3B, *P* < 0.05, n = 6–8). Furthermore, pre-treatment of cells with 10% CSE exacerbated (~2-fold) this hyperpermeability relative to vehicle-treated OGD + RO cells (Fig. 3B, *P* < 0.05, n = 8).

### Effect of CSE on superoxide and hydrogen peroxide levels in bEnd.3 cells

**Figure 4:**
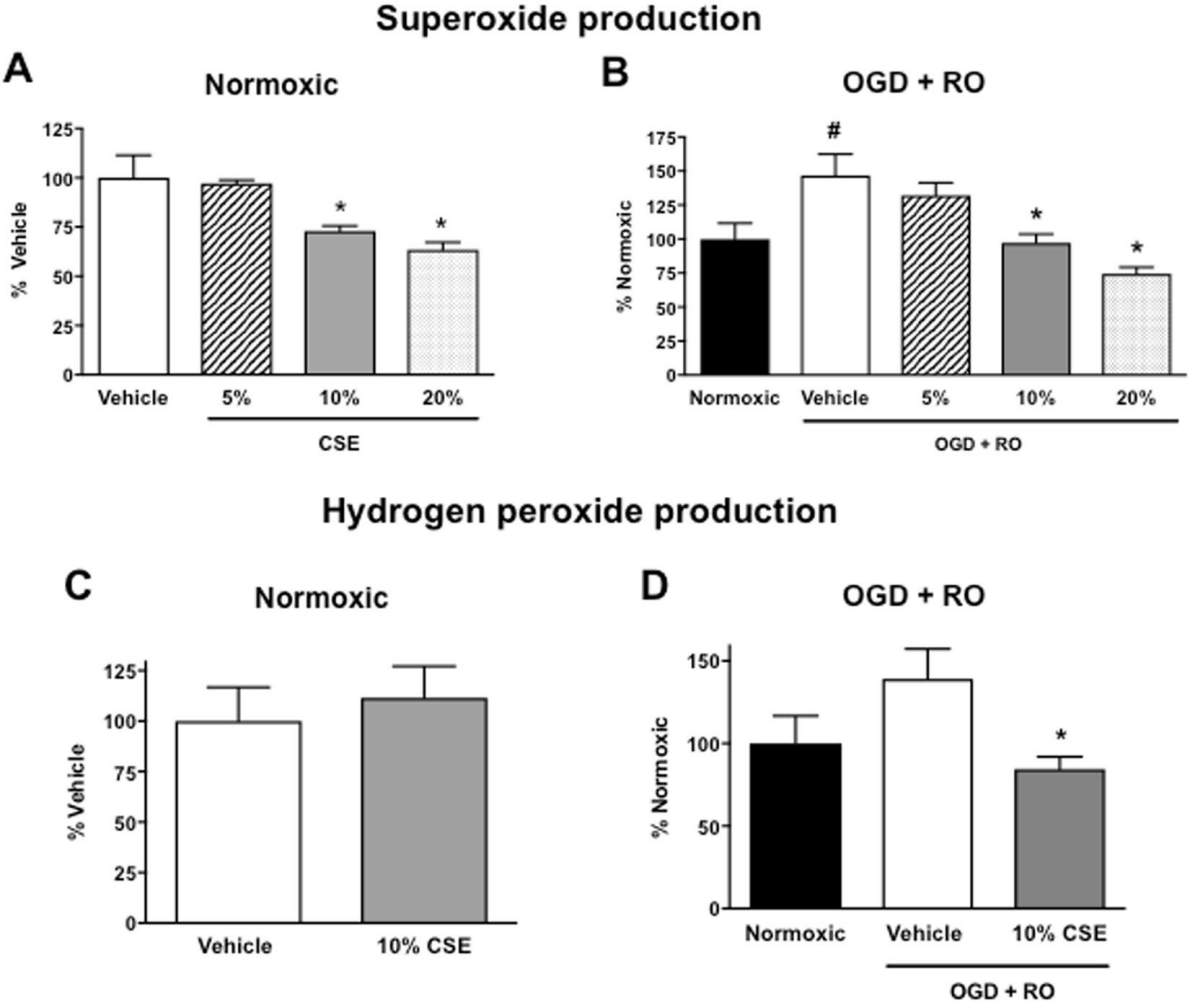
Effect of cigarette smoke extract (CSE: 5, 10, and 20%) on superoxide and hydrogen peroxide levels in bEnd.3 cells exposed to normoxic (**A**,**C**) or OGD + RO conditions (**B**,**D**). Results are expressed as a percentage of either vehicle-treated cells (A, C) or normoxic controls (B, D) and are given as mean ± SEM (n = 5–6). *P < 0.05 vs. vehicle-treated cells, ^#^*P* < 0.05 vs. normoxic controls (One-way ANOVA with Bonferroni post-hoc test).

Using L-012 chemiluminescence and the Amplex Red fluorescence assay, we next assessed whether the effect of CSE on BBB permeability is associated with alterations in the production of superoxide and/or hydrogen peroxide, which both influence BBB integrity. Treatment of normoxic bEnd.3 cells with 5% CSE had no significant effect on superoxide levels (Fig. 4A, *P* > 0.05, n = 5), whereas superoxide levels were significantly lower in cells treated with 10% or 20% CSE (Fig. 4A, *P* < 0.05, n = 6). Treatment of normoxic bEnd.3 cells with 10% CSE had no significant effect on hydrogen peroxide levels (Fig. 4C, *P* > 0.05, n = 5).

Consistent with our previous work^24^, superoxide levels in bEnd.3 cells were markedly elevated (~47%) after exposure to OGD + RO when compared with normoxic controls (Fig. 4B, *P* < 0.05, n = 6). Furthermore, there was a trend for hydrogen peroxide levels to also be elevated (Fig. 4D, *P* > 0.05, n = 5). By contrast, superoxide levels were not elevated after OGD + RO in cells treated with either 10% or 20%. In fact, superoxide levels were significantly lower compared with vehicle-treated OGD + RO cells (Fig. 4B, *P* < 0.05, n = 6). Hydrogen peroxide levels were also significantly lower in cells treated with 10% CSE relative to vehicle-treated OGD + RO cells (Fig. 4D, *P* < 0.05, n = 5).

### Effect of CSE on mRNA expression of Nox catalytic subunits, SOD, and Gpx-1

**Figure 5:**
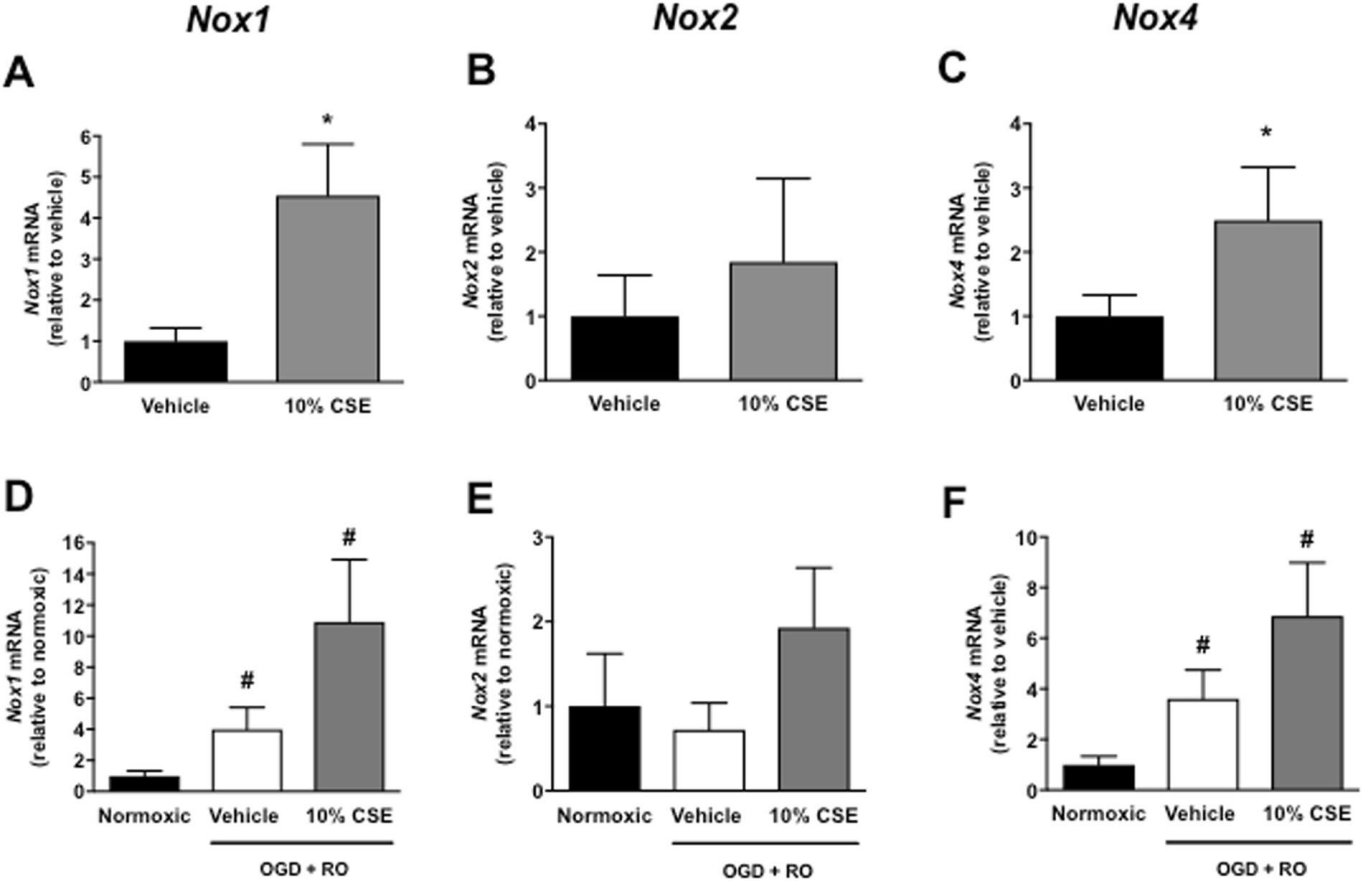
Effect of cigarette smoke extract (CSE: 10%) on mRNA expression of Nox1, Nox2, and Nox4 in bEnd.3 cells exposed to normoxic (**A**–**C**), or OGD + RO (**D**–**F**) conditions. Results are expressed as fold-change relative to either vehicle-treated cells (**A**–**C**) or normoxic controls (**D**–**F**) and are given as mean ± SEM (n = 8). *P < 0.05 vs. vehicle-treated cells (Student’s unpaired t-test), ^#^*P* < 0.05 vs. normoxic controls (One-way ANOVA with Bonferroni post-hoc test).

**Figure 6:**
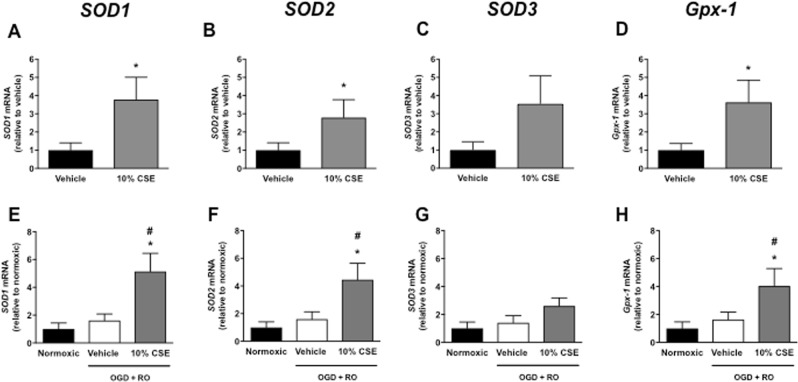
Effect of cigarette smoke extract (CSE: 10%) on mRNA expression of SOD1, SOD2, SOD3, and Gpx-1 in bEnd.3 cells exposed to normoxic (**A**–**D**), or OGD + RO (**E**–**H**) conditions. Results are expressed as fold-change relative to either vehicle-treated cells (**A**–**C**) or normoxic controls (**D**–**F**) and are given as mean ± SEM (n = 8). *P < 0.05 vs. vehicle-treated cells (**A**–**D**, Student’s unpaired t-test; (**E**–**H**), One-way ANOVA with Bonferroni post-hoc test). ^#^*P* < 0.05 vs. normoxic controls (One-way ANOVA with Bonferroni post-hoc test).

Using RT-PCR, we next assessed whether the effect of CSE on ROS levels is associated with altered expression levels of Nox oxidase catalytic subunits (Nox1, Nox2, and Nox4) and/or antioxidant enzymes (SOD1-3, Gpx-1). 10% CSE significantly increased Nox1, Nox4, SOD1, SOD2, and Gpx-1 mRNA expression in normoxic bEnd.3 cells relative to vehicle-treated cells (Figs 5A,C, 6A,B,D, *P* < 0.05, n = 8), whereas Nox2 and SOD3 expression levels were comparable between the two groups (Figs 5B & 6C, *P* > 0.05, n = 8).

Exposure of bEnd.3 cells to OGD + RO increased Nox1 and Nox4 mRNA expression relative to normoxic controls (Fig. 5D,F, *P* < 0.05, n = 8), but had no significant effect on expression levels of the SODs and Gpx-1 (Fig. 6E–H, *P* > 0.05, n = 8). 10% CSE significantly increased Nox1 and Nox4 expression in OGD + RO cells relative to normoxic controls, and there was a trend for an increase in expression levels of the three Nox isoforms relative to vehicle-treated OGD + RO cells (Fig. 5D–F, *P* > 0.05, n = 8). 10% CSE also significantly increased expression of SOD1, SOD2, and Gpx-1 in OGD + RO cells relative to vehicle treated normoxic, and OGD + RO cells (Fig. 6E,F,H, *P* < 0.05, n = 8). By contrast, Nox2 and SOD3 expression were comparable between groups (Figs 5E and 6G, *P* > 0.05, n = 8).

### Effect of CSE on mRNA expression of inflammatory cytokines

**Figure 7:**
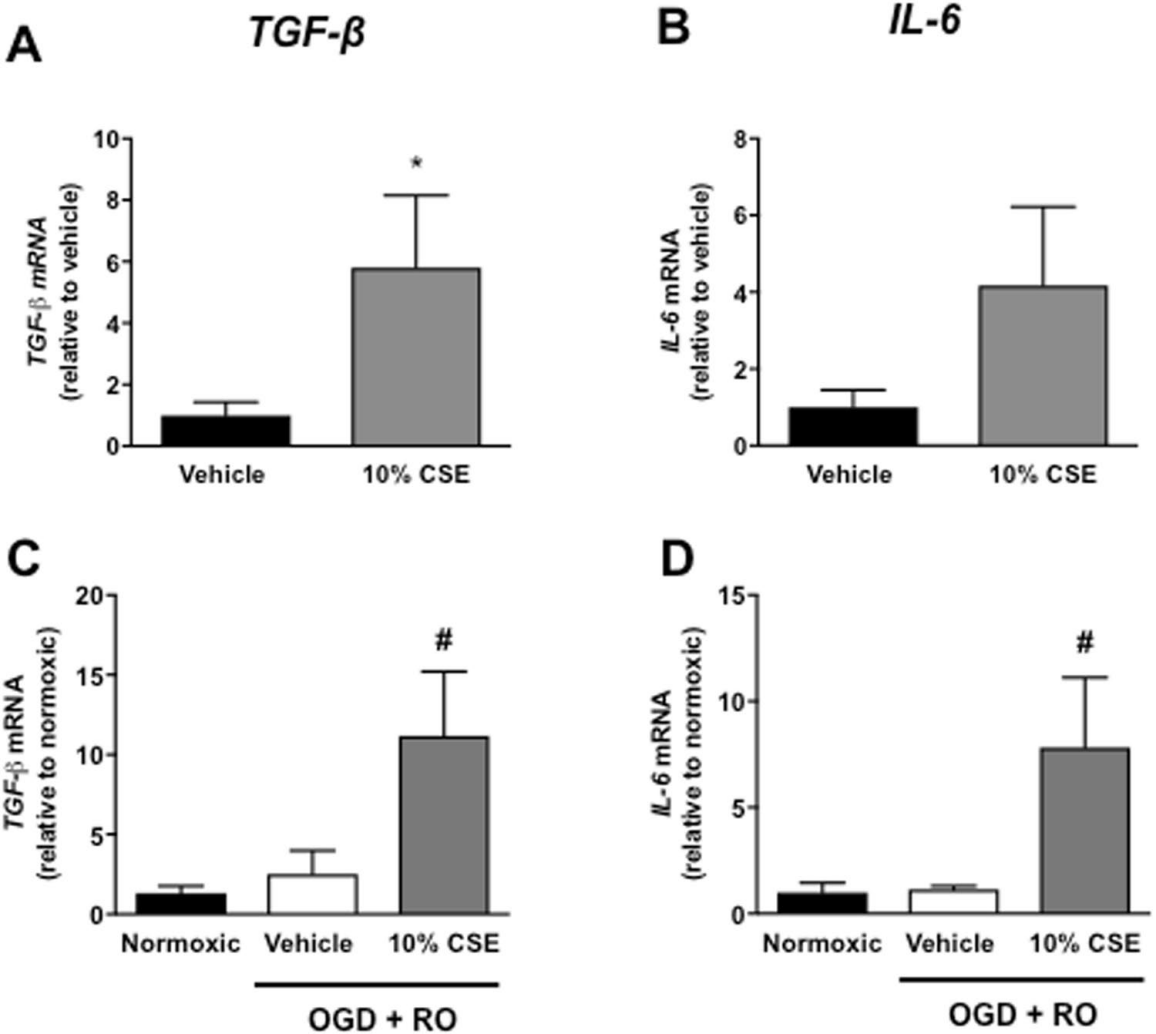
Effect of cigarette smoke extract (CSE: 10%) on mRNA expression of TGF-β and IL-6 in bEnd.3 cells exposed to normoxic (**A**,**B**), or OGD + RO (**C**,**D**) conditions. Results are expressed as fold-change relative to either vehicle-treated cells (**A**,**B**) or normoxic controls (**C**,**D**) and are given as mean ± SEM (n = 6). *P < 0.05 vs. vehicle-treated cells (Student’s unpaired t-test), ^#^*P* < 0.05 vs. normoxic controls (One-way ANOVA with Bonferroni post-hoc test).

CSE (10%) caused a significant increase in TGF-β mRNA expression (Fig. 7A) and a trend for an increase in IL-6 mRNA expression (Fig. 7B) in normoxic cells. However, CSE significantly increased TGF-β and IL-6 mRNA expression in OGD + RO cells (Fig. 7C,D, *P* < 0.05, n = 6). IL-4, IL-1β, TNF-α, and IFN-γ mRNA were undetectable in all normoxic and OGD + RO experimental groups (data not shown).

## Discussion

This study aimed to determine whether CSE worsens ischaemia-induced BBB disruption *in vitro* by triggering oxidative stress and/or inflammation. We found that the loss of BBB integrity following ischaemic/reperfusion-like conditions (OGD + RO) was indeed significantly worsened by CSE; however, contrary to our hypothesis this was not associated with elevated ROS levels. In fact, levels of superoxide and hydrogen peroxide were markedly lower in response to CSE, possibly because of upregulated antioxidant defence. Lastly, CSE increased the expression of pro-inflammatory cytokines in cerebral endothelial cells exposed to ischaemic/reperfusion-like conditions. Collectively, these results suggest that acute cigarette smoke exposure worsens BBB disruption after stroke potentially via inflammatory mechanisms. Whilst *in vivo* studies are needed, these findings raise the possibility that the increased stroke risk and severity in smokers (and potentially COPD patients) might relate, at least in part, to the deleterious effect of cigarette smoke on BBB integrity.

Cigarette smoking is a risk factor for stroke and is linked to worse stroke outcomes^27^. Findings from this *in vitro* study and previously published work^17,19,20^ show that CSE, which contain the soluble components of cigarette smoke, causes dysfunction of the cerebrovascular endothelium (in the absence of ischaemia-like conditions). Indeed, using a concentration of CSE that does not induce overt endothelial cell death, we show here that acute exposure of monolayers of normoxic cerebral endothelial cells to CSE markedly increased paracellular permeability, indicative of BBB disruption. These findings are consistent with studies showing that CSE causes a loss of BBB integrity in monolayers of human cerebral endothelial cells (hCMEC/D3) in association with altered expression of tight junction proteins (e.g. ZO-1, occludin, VE-cadherin and claudin-5)^17,19,20^. Cerebral endothelial dysfunction (particularly at the level of the BBB) contributes to and exacerbates ischaemic brain injury^28^. Therefore, the primary focus of this study was to test whether cigarette smoking influences stroke pathogenesis, at least in part, by worsening ischaemia-induced BBB disruption. We tested this using a well characterised *in vitro* BBB stroke model, which produces many of the key features of BBB disruption *in vivo* including paracellular hyperpermeability, tight junction protein degradation, and MMP-expression^23,24^. Indeed, in this study we found that exposure of cerebral endothelial cells to OGD + RO caused a marked loss of BBB integrity. The new finding from these experiments however was that the BBB disruption following ischaemic/reperfusion-like conditions was significantly worsened by prior treatment with CSE. Together these results strengthen evidence that acute cigarette smoking triggers BBB disruption *per se*, and show for the first time that this in turn might exacerbate the loss of BBB function and integrity after stroke.

Oxidative stress and inflammation both contribute to the complex mechanisms of BBB disruption after cerebral ischaemia and reperfusion. Cigarette smoke contains over 4000 chemical compounds including ROS, reactive nitrogen species (RNS), nitric oxide (NO), and free radicals of organic compounds^11^. In addition to these relatively short-lived ROS/RNS, cigarette smoke contains more stable substances that have the potential to increase ROS production including saturated and unsaturated aldehydes, unsaturated ketones, and nicotine^29,30^. Some of these compounds have been shown to react with thiol groups involved in the regulation of enzymes such as the ROS generating Nox oxidases^29,30^. In addition to triggering oxidative stress and inflammation in the lungs, oxidants and more stable compounds can pass into the circulation and initiate oxidative stress and inflammation in cells and tissues remote from the primary site of exposure^6^. Indeed, previous studies have shown that CSE elevates ROS levels in various cell types (e.g. neurons, glial, and endothelial and vascular smooth muscle cells of systemic origin) via Nox oxidase activation, enzymes that a major sources of ROS in cerebral vessels^25,31,32^. We therefore postulated that CSE worsens BBB disruption after OGD + RO by augmenting ROS levels and/or triggering an inflammatory response. Contrary to our hypothesis, although superoxide levels were elevated in control cells after OGD + RO, we found that prior treatment with CSE did not elevate levels further. In fact, despite evidence for increased gene expression of Nox1 and Nox4 catalytic subunits, superoxide levels in CSE treated cells after OGD + RO were in fact ~50% lower than controls. Consistent with this lower level of superoxide, hydrogen peroxide (the downstream metabolite of superoxide) levels were similarly lower in CSE treated cells after OGD + RO. Thus, these *in vitro* findings suggest that cigarette smoke exposure does not trigger or worsen BBB disruption by elevating ROS levels in cerebral endothelial cells.

Some studies have shown that antioxidant defence mechanisms are upregulated in endothelial cells in response to exposure to CSE. For example, acute exposure of human cerebral endothelial cells to CSE upregulates the expression of nuclear-factor (erythroid derived 2) related factor-2 (Nrf2)^19^. Nrf2 is one of the major transcription factors regulating the antioxidant defence response to oxidative stress, including the upregulation of the enzymes responsible for ROS metabolism (SODs, catalase, and Gpx1)^33^. Consistent with this, we found evidence of increased expression of SOD1, SOD2, and Gpx1 in response to CSE. We propose that upregulation of these antioxidant enzymes occurs in response to, and to counteract the oxidative stress caused by cigarette smoking. It remains to be determined, however, if this protection is sustained over longer periods of cigarette smoke exposure or whether the oxidative stimuli in cigarette smoke overcomes the protective antioxidant mechanisms of cerebral endothelial cells.

We then went on to explore whether the worsening of CSE-induced BBB disruption was associated with increased inflammatory gene expression. We found that IL-4, IL-1β, TNF-α, and IFN-γ mRNA were all undetectable. Similarly, a study found that IL-1β and TNF-α levels were below the level of detection (ELISA) in hCMEC/D3 cells following exposure to CSE^34^. In contrast to these cytokines, we found that IL-6 and TGF-β were significantly greater in CSE-treated cerebral endothelial cells after OGD + RO. However, whether this translates to increased cytokine levels remains to be determined. Furthermore, future experiments should be performed to determine whether elevated gene expression of IL-6 and TGF-β in response to CSE leads to increased cytokine-mediated signaling, e.g. phosphorylation of TGF-β receptor-regulated Smad proteins. Our findings are however consistent with previous studies showing that CSE promotes cytokine production (e.g. IL-6, IL-8, TGF-β) and the expression of adhesion molecules (e.g. vascular endothelial adhesion molecule-1, platelet endothelial cell adhesion molecule-1) in endothelial cells^19,34^. The roles of IL-6 and TGF-β in ischaemic stroke and BBB disruption are however not fully elucidated. IL-6 and TGF-β have been shown to increase the paracellular permeability of monolayers of cerebral endothelial cells *in vitro* by triggering a loss of tight junction proteins^35,36^. Consistent with this, inhibiting the effects of IL-6 with neutralizing antibodies attenuates ischaemia-induced BBB disruption in a hypoxic-ischaemic brain injury model^37^. By contrast, IL-6 has been reported to support the integrity of the BBB in a rat model of cerebral ischaemia^38^. Thus, future studies are needed to fully determine whether these cytokines contribute to the deleterious effects of cigarette smoke exposure on BBB integrity.

This study used a well-characterised and utilized immortalised cerebral endothelial cell line which displays many of the key features of the BBB such as the expression of key tight junction proteins, as well as the expression of key endothelial cell markers^23^. Monolayers of bEnd.3 cells are known to exhibit moderate paracellular permeability to high molecular weight molecules and have TEER readings ranging from 100–140 Ω cm^2^ ^39,40^, which is similar to that of primary cultures from several species (50–180 Ω cm^2^)^41^. Furthermore, bEnd.3 cells have the advantage of being cost effective compared with immortalised human cell lines or primary cerebral endothelial cells. Nevertheless, primary cerebral endothelial cells have the closest similarity to the BBB phenotype *in vivo* at low passage numbers. Thus, future studies should be performed to validate our findings in monolayers of primary cerebral endothelial cells, and/or using an immortalised human cerebral endothelial cell line.

In summary, the study found that cigarette smoke exposure worsens ischaemia-induced BBB disruption. Whilst cigarette smoke exposure increased the gene expression of Nox oxidases this did not translate to elevated levels of either superoxide or its downstream metabolite hydrogen peroxide, possibly because of upregulated antioxidant defence. Future studies should examine whether this protection is sustained chronically or whether cigarette smoke exposure overcomes the protective antioxidant mechanisms of cerebral endothelial cells. Lastly, we found evidence of increased proinflammatory gene expression in response to cigarette smoke exposure, suggesting the involvement of inflammatory mechanisms. The deleterious effect of CSE on BBB integrity might be consistent with the occurrence of a biological process known as endothelial to mesenchymal transition (EndoMT). Similar to airway epithelial to mesenchymal transition (EMT), EndoMT is a complex process whereby endothelial cells lose their specific markers such as the adheren junction protein VE-cadherins^42,43^, which is important for maintaining BBB integrity. Under chronic oxidative damage and inflammation (notably TGF-β) EndoMT can be initiated^42,44^. EMT is an active process in smokers and COPD patients^45,46^. Moreover, there is evidence that EndoMT might also be active in the pulmonary vasculature during COPD^47^. Thus, future studies could explore whether CSE initiates EndoMT in the cerebral endothelium and whether such a process contributes to the deleterious effects of CSE on BBB integrity. In conclusion, although future *in vivo* studies are needed, our findings raise the possibility that the deleterious effect of cigarette smoke on BBB integrity may contribute to stroke risk and severity in smokers and potentially COPD patients.

## Data Availability

All data generated or analysed during this study are included in this published article.
